# Cultivating Future Advocates: Training College Students to Become Long-Term Care Ombudsmen

**DOI:** 10.1093/geroni/igaf122.1157

**Published:** 2025-12-31

**Authors:** Corinne Kyriacou, Craig Rustici

**Affiliations:** Hofstra University, Hempstead, New York, United States; Hofstra University, Hempstead, New York, United States

## Abstract

Long-term care ombudsmen play a crucial role in advocating for vulnerable populations, mediating conflicts, and facilitating communication among residents, families, and facility staff. However, there is a shortage of volunteers trained to serve in this role. To address this long-term care need, a pilot program was launched as a collaborative initiative between a university and a state department of aging. The goals of this initiative were to develop a pipeline of certified long-term care ombudsmen, and to enrich undergraduate education by providing hands-on training in issues related to aging, disabilities, long-term care, advocacy, and conflict resolution. A committee of faculty and representatives from the state worked together over the course of nine months to develop a strategy and assess student interest. This spring, twenty undergraduate students from diverse majors, and two continuing education students enrolled in a special topics course that integrated the ombudsman training curriculum – inclusive of in-field shadowing – with a broader educational foundation in aging and disability studies, long-term care and medical communication. Preliminary findings from pre/post surveys and journal assignments indicate that students are motivated by the idea of empowering vulnerable populations, that they have limited knowledge of long-term care, and that they are interested in developing advocacy skills. Preliminary findings from stakeholder interviews with faculty and state officials show support for the pilot as a model to fill gaps in the long-term care in two ways – directly, by increasing supply of ombudsmen and indirectly, by increasing awareness of aging and disability-related career paths.

